# Genome-Wide Analysis of the *FABP* Gene Family in Liver of Chicken (*Gallus gallus*): Identification, Dynamic Expression Profile, and Regulatory Mechanism

**DOI:** 10.3390/ijms20235948

**Published:** 2019-11-26

**Authors:** Zhang Wang, Ya-Xin Yue, Zi-Ming Liu, Li-Yu Yang, Hong Li, Zhuan-Jian Li, Guo-Xi Li, Yan-Bin Wang, Ya-Dong Tian, Xiang-Tao Kang, Xiao-Jun Liu

**Affiliations:** 1 College of Animal Science and Veterinary Medicine, Henan Agricultural University, Zhengzhou 450002, China; wangzh19930124@163.com (Z.W.); crescent0303@163.com (Y.-X.Y.); ziming0312@126.com (Z.-M.L.); m18738133860@163.com (L.-Y.Y.); lihong19871202@163.com (H.L.); lizhuanjian@163.com (Z.-J.L.); liguoxi0914@126.com (G.-X.L.); ybwang2008@henau.edu.cn (Y.-B.W.); ydtian111@163.com (Y.-D.T.); xtkang2001@263.net (X.-T.K.); 2 Henan Innovative Engineering Research Center of Poultry Germplasm Resource, Zhengzhou 450002, China; 3 International Joint Research Laboratory for Poultry Breeding of Henan, Zhengzhou 450002, China

**Keywords:** chicken, *FABP* gene family, liver lipid metabolism, estrogens, PPAR agonists

## Abstract

The fatty acid-binding protein (*FABP*) gene family, which encodes a group of fatty acid-trafficking molecules that affect cellular functions, has been studied extensively in mammals. However, little is known about the gene structure, expression profile, and regulatory mechanism of the gene family in chickens. In the present study, bioinformatics-based methods were used to identify the family members and investigate their evolutionary history and features of gene structure. Real-time PCR combined with in vivo and in vitro experiments were used to examine the spatiotemporal expression pattern, and explore the regulatory mechanism of *FABP* genes. The results show that nine members of the *FABP* gene family, which branched into two clusters and shared a conserved FATTYACIDBP domain, exist in the genome of chickens. Of these, seven *FABP* genes, including *FABP1*, *FABP3-7,* and *FABP10* were abundantly expressed in the liver of hens. The expression levels of *FABP1*, *FABP3*, and *FABP10* were significantly increased, *FABP5* and *FABP7* were significantly decreased, and *FABP4* and *FABP6* remained unchanged in hens at the peak laying stage in comparison to those at the pre-laying stage. Transcription of *FABP1* and *FABP3* were activated by estrogen via estrogen receptor (ER) α, whilst *FABP10* was activated by estrogen via ERβ. Meanwhile, the expression of *FABP1* was regulated by peroxisome proliferator activated receptor (PPAR) isoforms, of which tested PPARα and PPARβ agonists significantly inhibited the expression of *FABP1*, while tested PPARγ agonists significantly increased the expression of *FABP1*, but downregulated it when the concentration of the PPARγ agonist reached 100 nM. The expression of *FABP3* was upregulated via tested PPARβ and PPARγ agonists, and the expression of *FABP7* was selectively promoted via PPARγ. The expression of *FABP10* was activated by all of the three tested PPAR agonists, but the expression of *FABP4-6* was not affected by any of the PPAR agonists. In conclusion, members of the *FABP* gene family in chickens shared similar functional domains, gene structures, and evolutionary histories with mammalian species, but exhibited varying expression profiles and regulatory mechanisms. The results provide a valuable resource for better understanding the biological functions of individual *FABP* genes in chickens.

## 1. Introduction

Fatty acids (FAs) serve in fat synthesis, assembly, and storage in different cellular substructures including the mitochondria, peroxisome, endoplasmic reticulum, lipid droplet, and nucleus [1]. Their hydrophobic nature requires carrier protein assistance for FA transport.The *FABP* gene family can bind long-chain fatty acids (LCFAs) (C12-20), suppress the stain remover-like properties of FA, and traffic these ligands to various cellular compartments [2]. Several functions of *FABP* genes have been described: (a) mediating the anabolism or catabolism of lipid metabolic pathways; (b) maintaining levels of intracellular fatty acid; and (c) regulating the transcription of FA-responsive genes [3,4].

The *FABP* gene family is one category of the intracellular lipid-binding protein (iLBP) superfamily, which also includes cellular retinol-binding proteins (CRBPs) and cellular retinoic acid-binding proteins (CRABPs) subfamilies [3]. Atotal of 12 *FABP* genes have been identified in vertebrates until now, but not all members of *FABP* genes occur in the same species [4]. For example, *FABP10* and *FABP11* have only been proposed in nonmammalian vertebrates, like teleost fishes [5], while *FABP12* appears restricted to mammals, such as human [6]. All vertebrate *FABP* genes possess four exons separated by three introns [7], and contain a classical three-element finger print domain shared by three motifs termed FATTYACIDBP 1-3 (Kyoto Encyclopedia of Genes and Genomes; PRINTS: PR00178) [8]. Although these motifs have a low homology, they still share a similar β-barrel tertiary structure, which is designed to provide an internal cavity that serves as a binding site for hydrophobic ligands [9]. In mammals, as an enhanced adaptation of genes to the environment, diverse *FABP* genes may have evolved to enable cytoplasmic transport of distinct ligands [10]. The *FABP* family is derived from a single ancestral gene encoding a lipid-binding protein and forms three clusters, according to phylogenetic analysis [11]. Cluster 1 includes *FABP1* and *FABP6*, whose proteins bind hydrophobic groups such as heme, acyl-CoA, and bile acids (BAs). Cluster 2, the largest subfamily, includes *FABP3*, *FABP4*, *FABP5*, *FABP7*, *FABP8,* and *FABP9*, whose products bind LCFAs, eicosanoids and retinoids [12]. Cluster 3 only contains *FABP2*, whose product binds LCFAs alone. Most FABP proteins show high-affinity binding of hydrophobic ligands in a 1:1 stoichiometry, though FABP1 can bind these bulkier ligands and accommodate up to two LCFA molecules per protein molecule due to its larger cavity volume [13,14]. In addition, *FABP* genes can protect eicosanoid intermediates against peroxidation via binding these substrates, suggesting antioxidant-type behavior in mice [15]. These results indicate that *FABP*s play diverse roles in regulating metabolism. Indeed, studies with *FABP1*-null mice show that the stability of fatty acids in the liver decreases rapidly, implying that *FABP1* acts as the transporter mediating the anabolism of FAs [16,17]. Studies on *FABP4* support a role in the triacylglycerol (TG) storage of adipose tissue in chicken [18]. In addition, under high-fat diet conditions, male *FABP2*-null mice exhibited increased hepatic triacylglycerol (TG) deposition, as compared to corresponding wild-type mice, which may be associated with the specific role of *FABP2* in intestinal TG synthesis and/or transport [19].

Each *FABP* gene exhibits specific expression patterns of tissue, but they are expressed most abundantly in tissues involved in tissue-specific coordinated lipid responses, such as liver, adipose, and small intestine, where fatty acids are major materials for lipid metabolism [12,20]. The liver is the major site of fatty acid synthesis and transport, however, and compared with other *FABPs*, *FABP1* is the only one expressed abundantly in rat livers [21]. The small intestine is the site of the assimilation of dietary lipids via the enterohepatic circulation, where *FABP1* and the *FABP2* are expressed at high levels during mouse enterocyte differentiation [22]. It is clear that no *FABP* is specifically expressed in a single tissue and most tissues express several *FABP* isoforms, but the regulatory mechanisms of the tissue-specific expression and biological functions of various *FABP* genes is poorly understood.

Previous studies have reported nine separate *FABP* genes in the chicken genome, namely, *FABP1-8* and *FABP10* [23]. Studies on *FABP1*(also known as *L-FABP*) and *FABP10* (also known as *LbFABP*) have revealed that *FABP1* is highly expressed in chickenlivers and intestines, whereas *FABP10* is specifically expressed in livers [24]. Further investigations suggest that these two genes’ expression is associated with abdominal fat weight in chickens [25,26]. In addition, northern blot analysis indicates that *FABP3* (also known as *H-FABP*) is expressed in a wide variety of chicken tissues, and *FABP4* (also known as *A-FABP*) is mainly expressed in adipose tissue [18,27]. The mRNA level of *FABP4* in adipose tissue is significantly correlated with intramuscular fat in BeijingYou chicken and Jingxing chicken [28]. No more detailed characteristics of the temporal or spatial expression of the *FABP* family are known in chickens.

It has been demonstrated that transcriptional regulation of *FABP* genes is associated with the PPAR signal pathway [29]. The PPARα-mediated signaling pathway accelerates β-oxidation in human livers [29], PPARγ plays an important role in lipid storage in chicken adipose tissue [30], and PPARβ is known to regulate chicken fat deposition [31]. PPAR transcription factors affect lipid storage and metabolism by regulating downstream target genes that have functional peroxisome proliferator response element (PPRE) sites on their promoters. The PPRE site is made up of a relatively poorly conserved 5′ flanking region (5′FR) (underlined: 5′-CAAAACAGGTCANAGGTCA-3′) and a conserved direct repeat element (DR1) (underlined: 5′-CAAAACAGGTCANAGGTCA-3′) [32]. DR1 sequences affinity the PPAR complex directly, and 5′FR may distinguish the different PPAR subforms selectively [33]. Previous studies describe that ligands such as fatty acids or other hydrophobic agonists bind these transcription factors, and control the transcription of *FABP1*, *FABP3*, *FABP4* and *FABP5* in C2C12 myoblasts and mouse keratinocytes [34,35]. PPARs may interact with NF-κB or AP-1 molecules to facilitate repression of gene transcription in immune system [36]. In addition, FABP proteins can bind FA to activate PPAR and retinoid X receptor (RXR) heterodimers in the nucleus [34], suggesting feedback in the FA-PPAR-FABP pathway. Whether and how PPARs regulate the *FABP* genes in chickens is not known.

The liver is extremely important in lipid metabolism in chickens, and the progression of lipid metabolism is largely regulated by estrogen [37]. With sexual maturity of the hen, the plasma estrogen level reaches a peak and then gradually declines, but remains higher during the peak laying period [38,39,40]. Mechanistically, estrogens dimerize with their nuclear hormone receptors, including ERα and ERβ in the cellular cytosol in mice [41,42]. Thereafter, the dimers activate the transcription of their target genes directly or indirectly by binding classical estrogen response elements (ERE) [43]. Previous studies demonstrate that the expression of the acyl-CoA synthetase family 1 (*ACSF1*) gene was upregulated directly by ERα in chickens [44]. The cathepsin E-A-like gene increases during the sexual maturation of chicken, suggesting that the regulatory effect is predominantly mediated through ERβ in the livers of chickens [45]. In addition, ER would change the gene expression when an AP1 site occurs in its promoters [46,47]. Whether and how estrogen regulates the expression of *FABP* genes in chickens is not yet known.

In the current study, protein motif composition, gene organization, and the evolutionary history of the *FABP* family in chickens were analyzed comprehensively. The expression profiles and regulatory mechanisms were explored (Supplementary Figure S1). Our findings have paved the way for further functional characterization of *FABP* gene family in chicken.

## 2. Results

### 2.1. Identification and Domain Analysis of FABP Genes in Chicken

**Figure 1:**
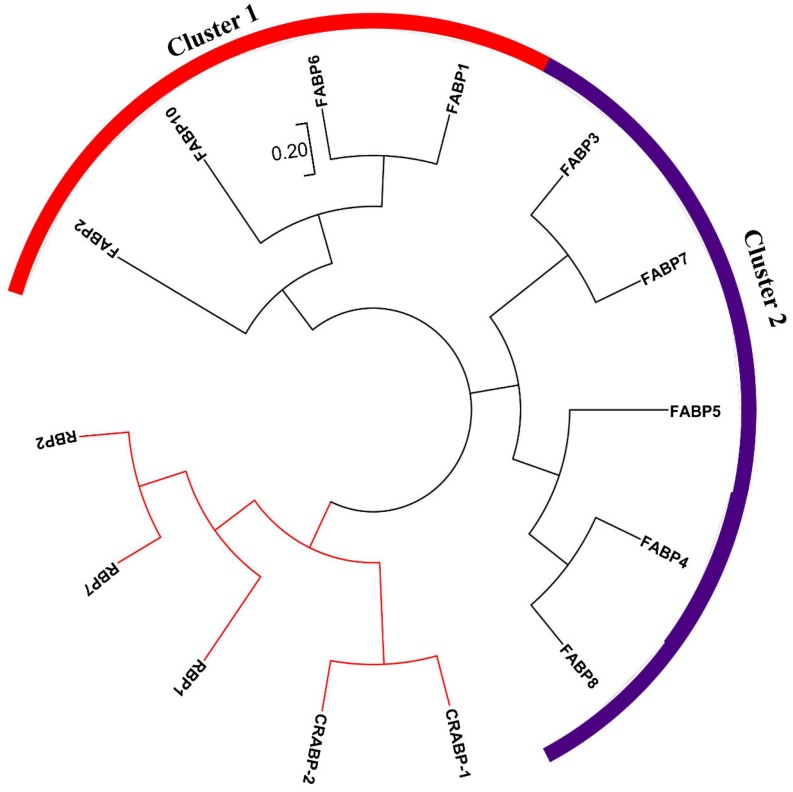
Molecular phylogenetic analysis of chicken iBP superfamily. The tree is drawn to scale, with branch lengths measured in the number of substitutions per site. *RBP* and *CRABP* genes were clustered from 14 amino acid sequences visualized as red branch. Chicken *FABP* genes were divided into two clusters according the phylogenetic relationship.

A total of fourteen protein sequences were acquired by HMM analysis and BLASTP against the protein sequences of known members of the FABP gene family (Figure 1). In order to distinguish different subfamilies of these protein sequences, an unrooted evolutionary tree was constructed to classify all of the sequences (Figure 1). The results suggest that two proteins, namely, CRABP1 (NP_001025710.1) and CRABP2 (XP_015153912.1), belong to the CRABP family, and three proteins; namely, RBP1 (NP_001264345.1), RBP2 (NP_001264346.1), and RBP7 (XP_417606.4), belong to the RBP family, with the five proteins clustered in a distinct group (Figure 1). Given that CRABPs, RBPs, and FABPs belong to a common iLBP superfamily, they share certain sequence similarities. Therefore, the two CRABP genes and three RBP genes were artificially removed from the list of fourteen putative chicken *FABP* genes. Finally, nine genes were identified in the chicken *FABP* gene family (Supplementary Table S1). The chicken *FABP* genes were grouped into two clusters according to their evolutionary relationships (Figure 1). Genomic location, amino acid (aa) number, molecular weight (MW), and isoelectric point (pI) are shown in Supplementary Table S1. Among the nine FABP proteins, FABP6 was identified to be the smallest (115 aa residues), whereas FABP5 was the largest (134 aa residues). The MW of the proteins ranged from 128.6 to 157.5 kDa, and the pI ranged from 5.33 (FABP6) to 8.53 (FABP10).

It is known that all FABP family members contain a conserved fingerprint domain, from teleost fishes to mammals (PRINTS pattern FATTYACIDBP; PR00178), which is divided into three motifs. In this study, the conserved FATTYACIDBP domain was also identified in chicken FABP proteins (Supplementary Table S2). The sequence similarity between the FATTYACIDBP1 sites ranged from 30.43% to 47.83%, whilst FATTYACIDBP2 ranged from 35.29% to 64.71%, and FATTYACIDBP3 from 22.73% to 40.91%. Further investigations found that the protein sequence similarity among members of the FABP family in chickens ranged from 25.37% to 39.37% (Supplementary Figure S2).

### 2.2. Phylogenetic Analysis and Gene Structure of Chicken FABP Genes

**Figure 2:**
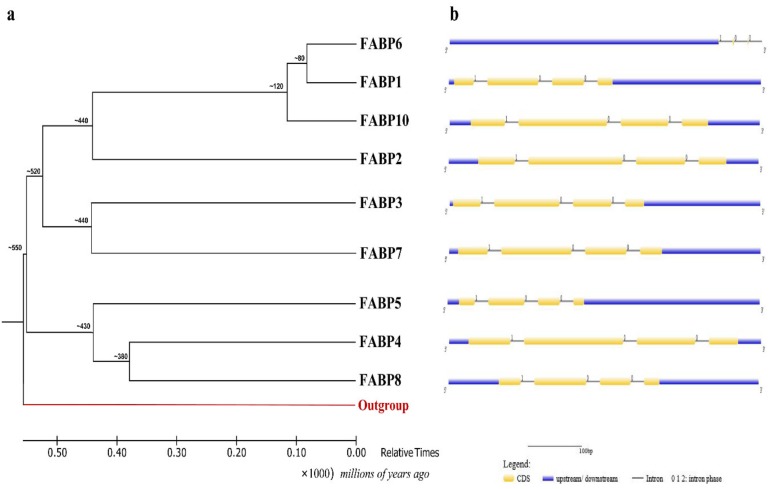
Phylogenetic relationship and gene structure analysis of chicken *FABP* genes: (**a**) Scheme for the evolution of the chicken *FABP* gene family. The tree was rooted by including an outgroup (*FABP11a*, Zebrafish) in the phylogenetic analysis. Gene duplication times are shown in millions of years ago and were estimated as described in the text; (**b**) Exon-intron structure of chicken *FABP* genes from untranslated 5′- and 3′-regions. Yellow boxes represent the exons which form the CDS, and the black lines represent the introns. The upstream/downstream highlighted by blue boxes. The number of 0, 1, and 2 are indicated splicing phase of intron.

To analyze the phylogenetic relationships of chicken *FABP* genes, the coding sequence (CDS)of chicken *FABP* members were retrieved from GenBank to construct a root tree. The location of the deepest branch (i.e., duplication of the primordial *FABP* gene) was determined by including a zebrafish *FABP11a* sequence (outgroup) during tree reconstruction (Figure 2a). Zebrafish *FABP11a* was estimated to have diverged approximately 700 MYA, in line with a previous study [48]. The divergence time between clusters 1 and 2 was estimated. The earliest chicken *FABP* gene duplication probably occurred at more than 550 MYA. Two clusters were estimated to have diverged approximately550 MYA. In cluster 1, *FABP2* diverged with other *FABPs* approximately 440 MYA. The results indicate that chicken *FABP* genes were grouped into two clusters. *FABP10*, *FABP1,* and *FABP6* emerged via a latest common ancestral gene approximately from 120 to 80 MYA.

The GFF database was used to investigate the gene structure of chicken *FABPs*. The visualized gene structure of chicken *FABP* genes with the CDS and untranslated region (UTR) at the genomic level is seen in Figure 2b. The conserved chicken *FABP* genes consisted of four exons and three introns, except *FABP6*, which had six exons (Supplementary Table S3). According to the GeneBank database, the number of amino acids was similar within every exon of the chicken *FABP* genes, but the intron length varied. Typically, the lengths were 24–33 amino acids encoded by exon 1, 57–58 amino acids by exon 2, 30–42 amino acids by exon 3 and 16–17 amino acids by exon 4 (Supplementary Table S3). *FABP6* possessed the longest 5’ UTR sequence and the smallest 3’ UTR (Figure 2b).

### 2.3. Tissue Distribution of Chicken FABP Genes

**Figure 3:**
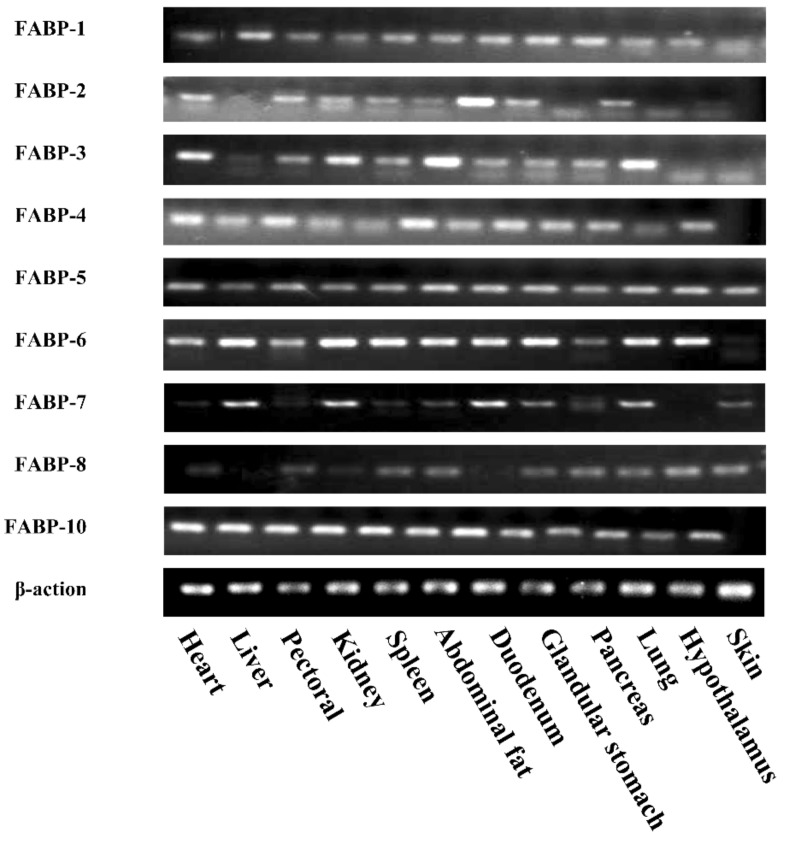
Tissue distribution of chicken *FABP* genes. The nine chicken *FABP* genes were semi-quantified by RT-PCR in twelve different chicken tissues.

To determine the expression pattern of *FABP* genes in chickens, cDNAs synthesized using RNAs isolated from 12 different tissues of 20-week-old Lushi chickens were used for PCR. It was shown that *FABP1*, *FABP6*, *FABP7,* and *FABP10* were highly expressed in the liver, while *FABP2* and *FABP8* were highly expressed in the glandular stomach and hypothalamus, respectively. The other members of the *FABP* gene family were universally expressed in various tissues (Figure 3).

### 2.4. Dynamic Expression Profiles of FABP Genes in Liver of Chicken

**Figure 4:**
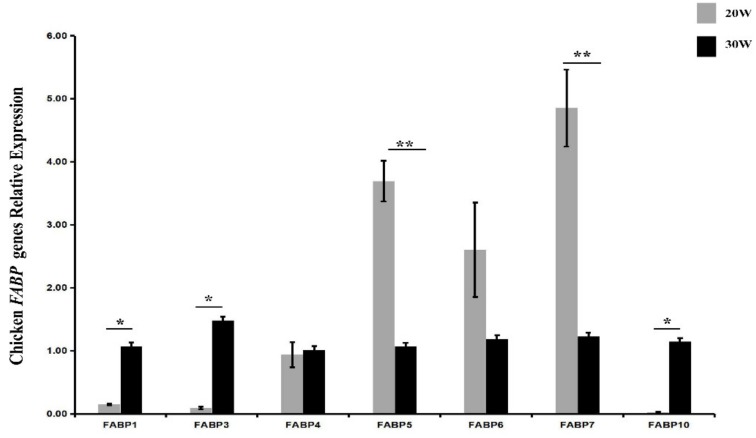
The expression profiles of chicken *FABP* genes in chicken liver between 20 and 30 weeks. Each value is represented the mean ± SE (*n* = 8). Student’s *t*-test was used to determine the statistical significance. * *p* < 0.05, ** *p* < 0.01.

The dynamic expression profiles of *FABP* genes in the liver were investigated by qPCR. The results showed different expression trends in the livers of pre-laying hens (20 weeks old) and peak-laying hens (30 weeks old). The expression levels of *FABP1*, *FABP3*, and *FABP10* increased with sexual maturation, while the expression levels of *FABP5* and *FABP7* significantly decreased, and *FABP2*, *FABP4*, and *FABP6* showed no changes (Figure 4).

### 2.5. Characteristics of Chicken FABP Promoters

To gain an insight into the regulatory mechanisms of the expression of *FABP* genes, 2 kb of upstream sequences from the TSS of *FABP* genes were retrieved, and putative EREs, AP-1, and PPREs were analyzed. The results indicate that a putative ERα binding site occurs in the promoter regions of the *FABP1* and *FABP3* genes, a putative ERβ site occurs in the promoter regions of the *FABP3* and *FABP10* genes, and a putative AP-1 site occurs in the promoter region of the *FABP5* gene (Table 1, Supplementary Figure S3). In addition, a putative PPRE was found in the *FABP1*, *FABP3*, *FABP5*, *FABP7*, and *FABP10* promoter regions (Table 2).

### 2.6. Effect of Estrogen on the Expression of Chicken FABP Genes In Vivo and In Vitro

**Figure 5:**
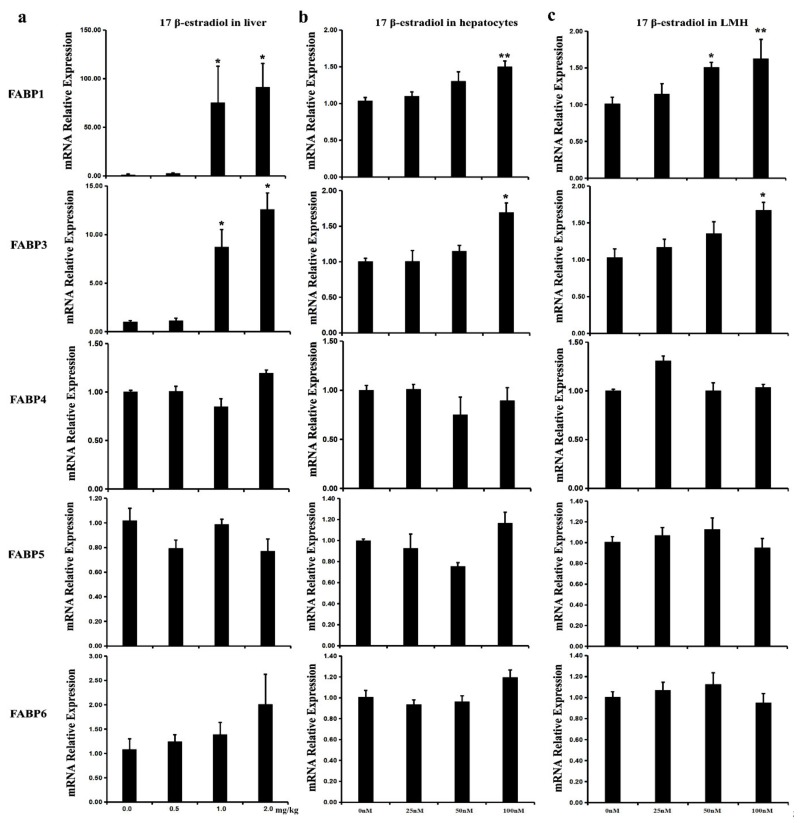

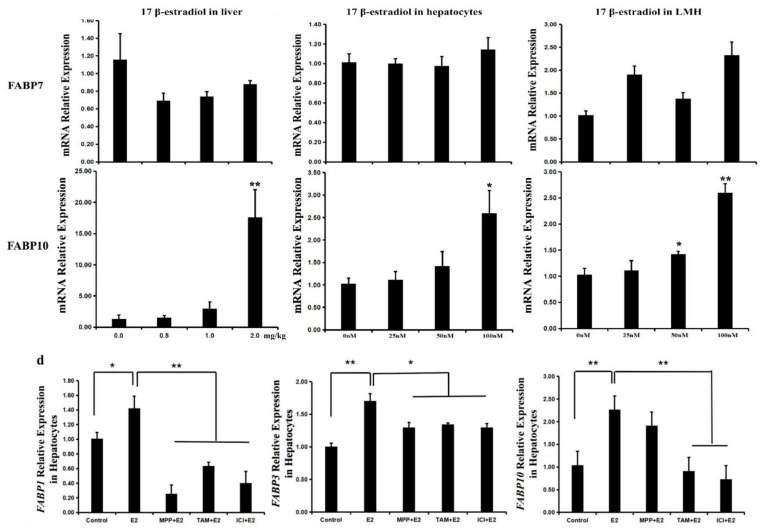
The expression of chicken *FABP*s were affected by 17β-estradiol treatment. The expression of seven *FABP* genes, including *FABP1*, *FABP3*, *FABP4*, *FABP5*, *FABP6*, *FABP7,* and *FABP10,* which expressed in chicken livers, were quantified by qPCR. (**a**) the effect of 17β-estradiol on seven *FABP* genes mRNA expression in chicken liver after different concentrations of estrogen treatment for 12 h; (**b**,**c**) The 17β-estradiol regulated in chicken primary hepatocytes and LMH cells after different concentrations of estrogen treated for 12 h; (**d**) ER antagonists inhibited the transcripts level of *FABP1*, *FABP3*, and *FABP10* by competing ERE sites with estrogen in chicken primary hepatocytes. The level of *FABP1*, *FABP3*, and *FABP10* were upregulated when 17β-estradiol treatment, and were partially inhibited by co-treated MPP, tamoxifen, or ICI 182, 780 with 17β-estradiol in chicken primary hepatocytes. E2:17β-estradiol (100nM); ICI: ICI 182,780 (1 μM), TAM: tamoxifen (1μM), and MPP: (1,3-bis(4-hydroxyphenyl)-4-methyl-5-[4-(2-piperidinylethoxy)phenol]-1*H*-pyrazoledihydrochloride) (1 μM). Each value is represented the mean ± SE (a, *n* = 10; b, c, d, *n* = 6). Student’s *t* test was used to determine the statistical significance. * *p* < 0.05, ** *p* < 0.01.

To verify the effect of estrogenon the transcriptional regulation of *FABP* genes in vivo, the expression levels of *FABP* genes in the liver of chickens treated with 17β-estradiol were analyzed using qPCR. The results show that the mRNA levels of *FABP1*, *FABP3*, and *FABP10* were significantly up-regulated in the liver of treated groups in comparison to that in the control group (Figure 5a). This result was further confirmed in chicken primary hepatocytes and LMH cells treated with 17β-estradiol (Figure 5b,c). The transcriptional levels of the other *FABP* genes were unchanged under 17β-estradiol treatment.

To further define the unique ER sub-forms that mediate estrogen’s effect on the expression of the *FABP1*, *FABP3*, and *FABP10* genes, chicken primary hepatocytes were co-treated with 17β-estradiol and specific ER antagonists, respectively. The expression levels of *FABP1* and *FABP3* were significantly inhibited when the cells were cotreated with 17β-estradiol and either MPP, TAM, or ICI (Figure 5d), whereas *FABP10* mRNA was downregulated only when the cells were co-treated with either TAM or ICI (Figure 5d). The results suggest that 17β-estradiol up-regulated the expression of both *FABP1* and *FABP3* via ERα, and *FABP10* via ERβ in chicken hepatocytes and LMH cells.

### 2.7. Effect of PPAR Agonists on the Expression of Chicken FABP Genes

**Figure 6:**
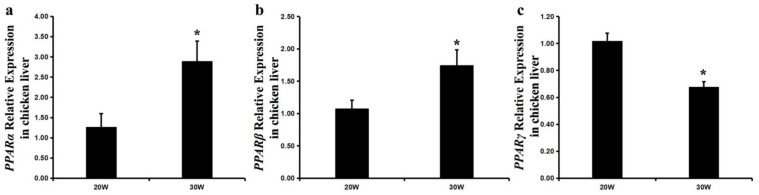
The expression profiles of chicken *PPARα*, *PPARβ*, and *PPARγ* genes in chicken livers between 20 and 30 weeks. Each value is represented the mean ± SE (*n* = 8). Student’s *t*-test was used to determine the statistical significance. * *p* < 0.05.

Previous studies report that the PPAR pathway is associated with the transcriptional activation of *FABP* genes. The expression profiles of *PPARα* and *PPARβ* show a significant increase, while expression level of *PPARγ* mRNA showed a decrease in the liver of 30 weekold peak-laying hens compared to 20 weekold pre-laying hens (Figure 6).

**Figure 7:**
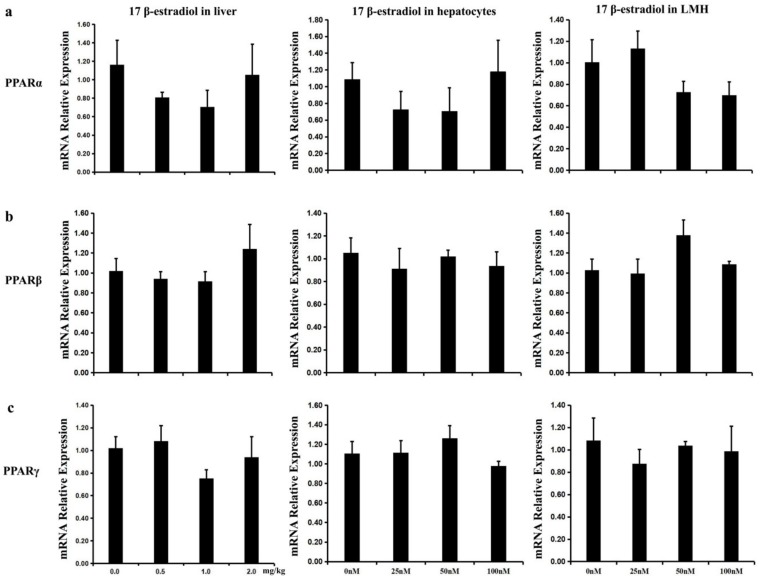
The expression of chicken *PPARs* were not activated by 17β-estradiol treatment in vivo and in vitro. The expression of three *PPAR* isoforms, including *PPARα*, *PPARβ* and *PPARγ* were quantified by qPCR. (**a**)The effect of 17β-estradiol on three *PPAR* isoforms mRNA expression in chicken liver after different concentrations of estrogen treatment for 12 h; (**b**,**c**) The expression of *PPAR* isoforms were not regulated by17β-estradiol in chicken primary hepatocytes and LMH cells after different concentrations of estrogen treated for 12 h. Each value is represented the mean ± SE (a, *n* = 10; b, c, *n* = 6). Student’s *t* test was used to determine the statistical significance.

To decipher the effects of 17β-estradiol on the mRNA expression of *PPAR* isoforms, RT-qPCR was performed to detect the expression of *PPAR* isoforms in estrogen-treated livers, hepatocyte, and LMH cell models. The results show that 17β-estradiol did not affect the mRNA levels of *PPAR* isoforms in vivo (Figure 7a) or in vitro (Figure 7b,c), indicating that individual PPAR isoforms exert their functions independent of estrogen.

**Figure 8:**
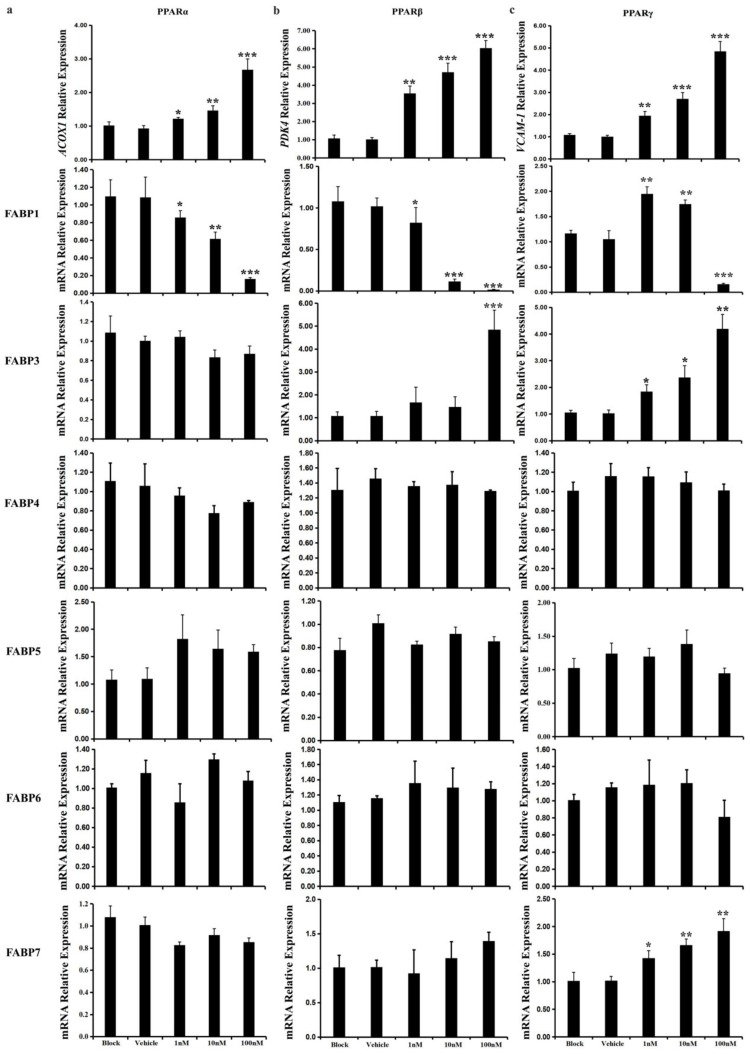

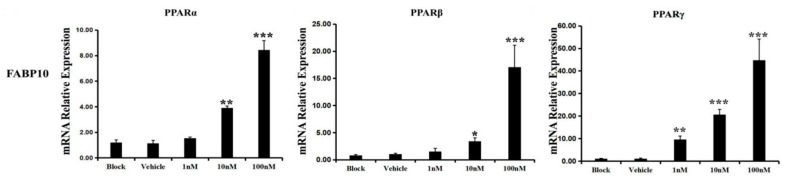
PPAR agonists regulated the expression of chicken *FABP* genes in LMH cells. The expression of seven FABP genes, including *FABP1*, *FABP3,FABP4*, *FABP5*, *FABP6,FABP7* and *FABP10* which expressed in chicken liver, were quantified by qPCR after WY14643, GW0742, or rosiglitazone treated for 24 h. The terminal concentration of PPAR agonists were 1 nM, 10 nM, and 100 nM, respectively. (**a**) The expression of *ACOX1* targeted by PPARα, were activated when treated by WY14, 643. The *FABP1* mRNA expressions were negatively regulated following the increasing dose of WY14, 643, while the *FABP10* level was increased; (**b**) The expression of *PDK4* targeted by PPARβ, were activated when treated by GW0742. The expression of *FABP1* was down-regulated whereas the transcripts level of *FABP3* and *FABP10* were promoted in a high terminal concertration; (**c**) The expression of *VCAM-1* targeted by PPARγ, were activated when treated by rosiglitazone. The expression of *FABP1* were upregulated in a lower dose of rosiglitazone, however, the effect were significantly reversed in a high concertration (100 nM). The transcripts level of *FABP3*, *FABP7*, and *FABP10* were upregulated. Each value is represented the mean ± SE (*n* = 6). Student’s *t* test was used to determine the statistical significance. * *p* < 0.05, ** *p* < 0.01, *** *p* < 0.001.

When the LMH cells were treated with different doses of the PPARα agonists WY14, 643 for 24 h, the expression level of acyl-coenzyme A oxidase 1 (*ACOX1*) was significantly increased, which showed a positive response to WY14,643 treatment [50] (Figure 8a). The mRNA levels of *FABP1* were significantly decreased, whereas *FABP10* appeared to have the reverse trend (Figure 8a). The transcriptional levels of the other *FABP* genes were unchanged under WY14,643.

When the LMH cells were treated with different doses of the PPARβ agonists GW0,742 for 24 h, the expression level of pyruvate dehydrogenase kinase-4 (*PDK4*), which has shown a positive response to GW0,742 treatment [51], was significantly increased. The expression of *FABP1* was significantly downregulated (Figure 8b). The transcriptional levels of *FABP3* and *FABP10* were increased (Figure 8b). The mRNA levels of the other *FABP* genes were unchanged after exposure to GW0,742.

Finally, when the LMH cells were treated with different doses of the PPARγ agonists rosiglitazone for 24 h, the expression level of vascular cell adhesion molecule 1 (*VCAM-1*), which has shown a positive response following rosiglitazone treatment [51]. The transcriptional level of *FABP1* mRNA in LMH cells treated with rosiglitazone at 1 or 10 nM for 24 h was significantly increased (Figure 8c). In contrast, higher concentrations of rosiglitazone (100 nM) significantly decreased *FABP1* lmRNA after 24 h (Figure 8c). The expression of *FABP3*, *FABP7,* and *FABP10* was significantly upregulated after exposure to rosiglitazone (Figure 8c). The transcriptional levels of the other *FABP* genes were unchanged.

## 3. Discussion

*FABP*s are known to bind free fatty acids and transport them to different organelles for lipid metabolism or storage [52]. To date, all vertebrates contain *FABP1*, *FABP2*, *FABP3*, *FABP6,* and *FABP7* genes among a total of twelve *FABP* genes [4]. In this study, fourteen different sequences were identified by initial search of chicken genome using human FABP protein sequences as queries. To accurately distinguish chicken *FABP* genes from these sequences, the unrooted evolutionary tree were constructed with fourteen protein sequences. The result demonstrated that nine *FABP* genes existed in chicken genome (Supplementary Table S1). Subsequently, a rooted tree was used to estimate the divergent time among nine members of chicken *FABPs*with CDS sequences. Similar to mammals [6], the sequences of the FATTPACIDBP domain show low homology among these nine chicken *FABP* genes. Seven chicken *FABP* genes, named *FABP1*, *FABP2*, *FABP3*, *FABP4*, *FABP5*, *FABP6*, *FABP7,* and *FABP8*, also present in the mammalian lineage, would have been inherited from their common ancestor. This *FABP* diversity likely arose from the two successive rounds of whole genome duplication (WGD) that occurred in early vertebrates [53,54]. In contrast, *FABP9* and *FABP12* appear restricted to mammals [55], but *FABP10* has been proposed in avian and teleost [56], revealing the relevance of *FABP* gene degeneration or duplication in the divergence of these chicken *FABP* genes from those of other vertebrates [23,53]. A previous study suggested *FABP10* originated before the most recent common ancestor (MRCA) of tetrapod and bony fish [23]. This viewpoint further illustrates that the *FABP10* gene in chicken is a copy of an ancestral *FABP* whose duplication occurred prior to the divergence of fish and tetrapod. Due to the occurrence of new WGD events and subsequent tandem duplication of *FABP* genes, more members formed in the *FABP* gene family [57]. The zebrafish have *FABP1a*, *FABP1b.1* and *FABP1b.2* and *FABP7a*/*FABP7b* [58]. Duplicated genes undergo non-functionalization, sub-functionalization or neo-functionalization, which may result in these paralogous genes appearing in different species [59]. A timescale is necessary for estimating rates of molecular change in organisms so we can interpret patterns of macroevolution, the molecular timescale of avians was estimated to begin 310 million years ago (MYA) [60]. In this study, the time tree showed that chicken *FABP6* and *FABP10* appeared posterior to teleost fish (~564 MYA) [60]. Duplications of other chicken *FABP*s may have occurred after the MRCA of mammals (synapsids) and birds (diapsids) diverged. These considerations may provide a further clear phylogenetic relationship of chicken *FABP* genes.

In the detection of male SD rats, *FABP*s are involved intracellular fatty acid transport in various tissues [61]. Therefore, basal expression cartography of *FABP* genes in chicken tissues was done for the first time in our study. Our semi quantitative results show that chicken *FABP*s exhibit tissue expression specificity. For example, *FABP2* and *FABP8* were not expressed in chicken liver, nor were *FABP4*, *FABP6* and *FABP10* in skin. Indeed, previous reports have shown that *FABP*s are selectively expressed in different tissues [62], among which *FABP2*, *FABP8*, and *FABP12* may exhibit tissue-specific expression patterns in intestine, medulla, and testis, respectively (in mammals) [63,64]. Compared with mammals, chicken *FABP*s are more widely expressed in various tissues, which seem to imply that *FABP*s play important roles in regulating metabolism, expected binding and trafficking hydrophobic ligands.

The liver is the main organ for lipid metabolism in chickens [37]. With the arrival of sexual maturity and the peak laying period, liver lipid metabolism is accelerated to meet the needs of egg yolk formation in laying hens [65]. It is generally agreed that estrogen plays vital roles in lipid metabolism in chickens [38]. In this study, we found that the expression levels of *FABP1*, *FABP3,* and *FABP10* were significantly increased, while the expression levels of *FABP5* and *FABP7* decreased from 20 weeks old (pre-laying stage) to 30 weeks old (peak laying stage). To explore whether the changes in the expression levels of *FABP*s between pre- and peak-laying hens were caused by changes of estrogen concentration, experiments in vivo and in vitro were set up. The results indicated that *FABP1*, *FABP3* and *FABP10* were significantly upregulated by 17β-estradiol administration. Further investigation showed that the effects of 17β-estradiol on promoting *FABP1* and *FABP3* expression could be partially inhibited by either the ER α antagonist MPP, or the ER α and ER β antagonists TAM and ICI 182,780, and the effects on promoting the expression of *FABP10* could only be repressed by TAM or ICI. It has been proven that MPP inhibits target genes by binding to ERα selectively [60]. TAM, as a synthetic estrogen antagonist, can repress the transcriptional activity of target genes via ERs [61]. ICI is a high-affinity estrogen receptor ligand for ERα and ER β [66]. Therefore, we inferred that the transcription of *FABP1* and *FABP3* were activated by estrogen via ERα, and that *FABP10* was activated by estrogen via ERβ (Supplementary Figure S4). Regarding the mechanism for the reduction of *FABP5* and *FABP7* expression levels during the peak-laying period in hens, further investigation is required. We speculate that *FABP1*, *FABP3,* and *FABP10* play key roles in exerting their functions in the liver in laying hens.

PPAR transcription factors regulate the expression of genes involved in lipid metabolism [67]. Previous studies suggested that differential expression patterns of PPAR-isoforms contribute to acquisition of differential expression of target gene, and these isoforms could bind to similar PPREs because the DNA-binding domain is the most conserved region among different PPAR isotypes [68,69]. However, study in zebrafish showed that PPAR transcription factors respond PPRE sites are preferentially bound by one of three PPAR isoforms [67]. In this study, we found that *FABP3* mRNA levels were positively affected byPPARβ and PPARγ agonists, the transcripts of *FABP7* were only activated by tested PPARγagonists, and *FABP10* responded positively to all tested PPAR agonists, but the expression levels of *FABP1* declined continuously as the concentrations of WY14,643 and GW0,742, agonists of PPARα and PPARβ, respectively, increased, agreeing with the previous study which concluded that tested PPAR-isoform agonists can specifically perform the actions mediated by PPARs and then upregulate the expression of target genes at the transcriptional level [33].Previous studies demonstrated that *PPARγ* mRNA level is reduced upon exposure to high concentrations of PPARγ agonists (>10 µM), including rosiglitazone and pioglitazonein HepG2 cells [70]. The results suggest that the expression of *FABP1* may be upregulated by tested PPARγagonists. In addition, though a PPRE was predicted in the *FABP5* promoter region, no effect of tested PPAR agonists on its expression was found. Taking all of the expression profiles of *FABP* genes and PPAR isoforms in the liver, between pre- and peak-laying hens, and the results of the PPAR-isoform agonist treatment experiments into consideration, we speculate that upregulated PPARα may increase the expression level of *FABP10*, PPARβ might contribute to the increase in the mRNA level of *FABP3* and *FABP10*, and PPARγ might contribute to the decrease in the expression level of *FABP7* in the livers of hens at the peak-laying stage (Supplementary Figure S4). However, whether PPARs are responsible for the increase the expression of FABPs still needs further study.

It has been previously reported that 17β-estradiol can interfere with PPARα-mediated actions, which leads to downregulation of the expression of genes targeted by PPARα in mice both differentiated C2C12 myotubes and skeletal muscle [71]. In addition, ER and PPAR can bind PPRE sites with high affinity, resulting in repression of genes targeted by PPARs [72]. For example, 17β-estradiol can attenuate the effects of fenofibrate to induce the expression of genes targeted by PPARα in mice [73]. However, our results imply that estrogen and PPARs independently exert their effects on the transcriptional regulation of *FABP* genes in the liver of chickens. The regulatory mechanisms of genes mediated by estrogen and PPARs remain to be studied.

## 4. Materials and Methods

### 4.1. Ethics Statement

The Animal Care Committee of Henan Agricultural University (Zhengzhou, China) approved this study (approval number 11-0085).

### 4.2. Identification and Classification of FABP Gene Family Members from the Chicken Genome

The chicken protein sequence database (GRCg6a. protein. fa) was downloaded from the National Center for Biotechnology Information Search database (NCBI, https://www.ncbi.nlm.nih.gov/genome/?term=CHICKEN). All twelve known FABP protein sequences were used as a query template (Supplementary Table S4) to build multiple alignment models according to domain similarity using hidden Markov model software (HMMER_build 3.0) to retrieve the possible chicken *FABP* proteins according to the default parameters [74]. In addition, a local Protein Basic Local Alignment Search Tool (BLASTP) analysis was used to identify the FABP with an e-value ≤ 1 × 10^−10^ in GRCg6a based on the same template protein sequences (Supplementary Table S4) [75].

In order to cluster different subfamilies of these sequences, all of the screened amino acidsequences were used to analysis phylogenetic relationship based on the maximum likelihood method by MEGA 7.0 to confirm them as members of the chicken *FABP* gene family [76].

### 4.3. Sequence Similarity and Domain Characterization

Alignment of *FABP* sequences from chicken was performed using ClustalW [77]. Percentage amino acid sequence identity and sequence similarity were determined using the BLOSUM62 matrix algorithm [78]. The conserved three-element fingerprint is a signature for all FABPs. To identify the FATTYACIDBP domains of chicken FABP, the sequences were submitted to the online software InterPro (https://www.ebi.ac.uk/interpro/beta/) with the default parameters to deduce amino acid sequences. The conserved three-element fingerprint domain FATTYACIDBP sequence of chicken FABPproteins was also characterized by ClustalW multiple-sequence alignment analysis with default parameters [77]. Lengths of sequences, molecular weights, and isoelectric points of identified FABP proteins were obtained from the ExPASy website (https://web.expasy.org/protparam/).

### 4.4. Phylogenetic Analysis and Annotation of Gene Structure of the Chicken FABP Gene Family

The phylogenetic trees were inferred using the maximum likelihood method in MEGA 7.0 with default parameters [79]. Zebrafish *FABP11a* (accession number NC_007130.7), whose gene duplication times have been estimated at ~679 to ~450 million years ago (MYA) [48], was used as an outgroup. The time-tree was generated using the RelTime method based on the CDS sequences of chicken *FABPs* [80]. Divergence times for branching points in the topology were calculated based on the JTT matrix-based model [81]. To determine the gene structure of chicken *FABP*s, the general feature format (GFF) database of chicken *FABP*s from NCBI was used as a reference. The visualization of exon-intron organization was acquired by TBtools software [82].

### 4.5. Predicted Regulatory Elements in the Promoter Regions of Chicken FABP Genes

The promoter region sequences of *FABP* genes were extracted within the 2.0 kb upstream of the *FABP* gene transcription start sites (TSS). The putative EREs (MA0112.3; MA0258.1), and PPREs (MA1148.1; MA0065.1) were acquired from the JASPAR vertebrate matrix group (http://jaspar.genereg.net/). Then, the promoter sequence was submitted to MEME FIMO to scan for individual matches to each ERE and PPRE (http://meme-suite.org/tools/fimo). The other regulatory elements were predicted by submitting sequences to JASPAR 5.0 online software (http://jaspar.genereg.net/) and visualized according to the operational guidelines of TBtools [82].

### 4.6. Animals, Estrogen Treatments, and Sampling

Sixteen female Lushi blue-shelled-egg chickens, raised in the same environment conditions, were killed at the age of 20 or 30 weeks old, respectively. Eight chickens were randomly selected for execution in each period. Tissues including heart, abdominal fat, liver, pectoral muscle, kidney, spleen, glandular stomach, duodenum, lung, pancreas, and adrenal glands were quickly removed, snap-frozen in liquid nitrogen and stored at −80 ℃ prior to use.

At 10 weeks old, 40 female Lushi blue-shelled-egg chickens were randomly divided into 4 groups, with 10 chickens in each group. The first three groups were experimental design groups and were injected intramuscularly with 0.5, 1.0, and 2.0 (mg/kg body weight) of 17 β-estradiol (Sigma, St. Louis, MO, USA) dissolved in olive oil, respectively. The control group of chickens was injected intramuscularly with the same volume of solvent (olive oil) only. All chickens were killed 12 h post injection with their livers snap-frozen in liquid nitrogen and stored at −80 ℃ until use.

### 4.7. Chicken Embryonic Primary Hepatocyte Culture and Treatments

Hepatocytes were isolated from the livers of chicken embryos incubated for 18 days according to the method described previously [83]. Briefly, fresh embryonic livers were shredded and washed to remove the impurities with PBS. Fragmented liver tissue was then digested by collagenase type V (Sigma) for 10 min and filtered by 500-, 200-, and 100-mesh filters. The hepatocytes were purified by non-continuous Percoll (Sigma) density-gradient centrifugation and washed three times with PBS.

Hepatocytes were suspended in Williams’ E complete medium (Sigma) containing 10% fetal calf serum (Gibco Corp., Carlsbad, CA, USA) to a concentration of 1 × 106 cells/mL, followed by plating into 12-well dishes. When cells had grown to 80–90% confluence, the medium was replaced with serum-free medium containing 100 U/mL penicillin and 100 mg/mL streptomycin solution and incubated for 6 h. The cells were then divided into four groups with three biological repetitions in each group. We treated the first three groups with 17β-estradiol at final concentrations of 25 nM, 50 nM, and 100 nM. The last group was treated with vehicle (0.1% ethanol) as a control. Finally, the cells were harvested 24 h later, and the expression of genes were detected by fluorescence real-time quantitative PCR (qPCR). The experiments were repeated three times independently.

It is known that 1,3-bis(4-hydroxyphenyl)-4-methyl-5-[4-(2-piperidinylethoxy) phenol]-1*H*-pyrazoledihydrochloride (MPP) is highly selective for ERα. Tamoxifen (TAM) and ICI 182,780 (ICI) (Sigma) are the primary antagonists for ERα and ERβ [84]. To further understand how estrogen regulates the expression of individual *FABP* genes, primary hepatocytes were divided into five groups with six replicates in each group. Cells were starved for 6 h when they grew to 80% confluence. Then, the first three groups were treated with 1 μM of ER subtype antagonists, MPP, TAM, and ICI (Sigma) dissolved in absolute ethanol. After 6 h, the cells were treated with 17β-estradiol at a final concentration of 100nM and harvested after 12 h. The cells in the fourth group served as controls and were treated with vehicle (0.1% absolute ethanol) alone for 6 h when they grew to 80% confluence and were then treated with 17β-estradiol at a final concentration of 100nM for 12 h. The gene mRNA levels were then detected by qPCR. The experiments were repeated three times.

### 4.8. Chicken LMH Cell Culture and Treatments

The LMH cell line was established from chicken primary hepatocellular carcinoma cells, which are a valuable tool to explore the chicken lipid mechanism of the liver [85]. The LMH cells were suspended in DMEM modified eagle medium (Gibco) with 1% penicillin and streptomycin solution and 10% fetal calf serum (Gibco) to a concentration of 1 × 10^5^ cells/mL, followed by plating into 12-well dishes. The treatment methods of 17 β-estradiol were similar to those for chicken embryonic primary hepatocytes mentioned above.

Each of the PPARα, -β, and -γ subtype antagonists, corresponding to WY14, 643 (Sigma), GW0, 742 (Sigma), and rosiglitazone (Sigma), were dissolved with 10 mg/mL in DMSO and then mixed with complete medium to a final concentration of 1 nM, 10 nM, and 100 nM, respectively. In order to establish whether and demonstrate how PPAR subforms regulate the expression of *FABP* genes, LMH cells were divided into eleven groups with six replicates in each group. When cells grew to 80% confluence, the first nine groups were treated with PPARα, -β, or -γ subtype antagonists at different concentrations. The cells in the tenth group served as a control group and were exposed to 0.5% DMSO, and the cells in the eleventh group served as a blank group and were cultured with complete medium throughout. The cells were harvested after 48 h of treatment, and changes in gene expression were detected by qPCR. The experiments were repeated three times independently.

### 4.9. RNA Extraction and Complementary DNA (cDNA) Synthesis

Total RNA was extracted from tissues and cells using Trizol reagent (Takara Bio Inc., Kyoto, Japan) according to the manufacturer’s protocol. The RNA integrity was determined by 1.5% denaturing agarose gel electrophoresis, and the purity and concentration were measured using NanoDrop2000 (Thermo Scientific, Wilmington, DE, USA). cDNA was synthesized with a PrimeScript™ RT reagent Kit (Takara Co. Ltd. Dalian, China) according to the manufacturer’s protocol and then stored at −20 °C until use.

### 4.10. Real-Time Quantitative PCR (RT-qPCR)

qPCR was conducted in a Roche LightCycler® 96 Instrument (Roche, CA, Switzerland) using TB Green™ Advantage® qPCR Premix (Takara, Kyoto, Japan). The qPCR primers of chicken *FABP*s and other genes were synthesized at Shanghai Sangon Biotech company (Shanghai, China) (Supplementary Table S5). qPCR was performed in a 10 μL reaction volume containing 1 μL of cDNA, 5 μL of TB Green RT-qPCR Mix, 0.5 μL of each forward and reward primer (10 μM each), and 4μL double-distilled water. The qPCR procedure was as follows: 95 °C for 30 s; 35 cycles at 95 °C for 5 s, 59.4 °C for 30 s, and 72 °C for 30 s; followed by 72 °C for 5 min. The housekeeping gene β-actin served as an internal control for normalization. All reactions were performed in triplicate. The relative gene expression was quantified using the comparative threshold cycle (2^−ΔΔCT^) method.

### 4.11. Statistical Analysis

All the experimental data are expressed as the mean ± SE and were processed using the statistical software SAS 9.1.3 (SAS Institute Inc., Raleigh, NC, USA). Statistical significance was determined using the *t*-test with SPSS version 23.0 (IBM, Chicago, IL, USA). *p* < 0.05 is considered a significant difference between groups.

## 5. Conclusions

A total of nine genes were identified in the chicken *FABP* gene family via genome-wide analysis. Phylogenetic analysis classified the *FABP* genes into two clusters with similar gene structures and conserved FATTYACIDB motifs. The expression patterns of the genes in different tissues implies that the *FABP* genes might play diverse roles in regulating lipid metabolism. Further bioinformatics analysis combined with in vivo and in vitro experiments demonstrate that estrogen and PPARs independently exert their effects on the transcriptional regulation of *FABP* genes in the liver of chicken. The increased expression levels of *FABP1*, *FABP3,* and *FABP10* in the liver of peak-laying hens is regulated by estrogen, and the decreased expression level of *FABP7*is regulated by PPARγ. Meanwhile, PPARγ might contribute to the increased expression levels of *FABP3*, and PPARα, -β and -γ contribute to the increased expression level of *FABP10* (Supplementary Figure S5). These results serve as a fundamental resource for better understanding the biological functions of individual *FABP* genes in chicken.

## Figures and Tables

**Table 1:** Putative ERE in chicken *FABP* genes promoter.

	ERα	ERβ
	Sequence	Sequence
**Consensus**	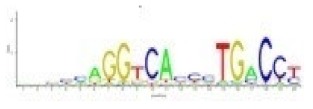	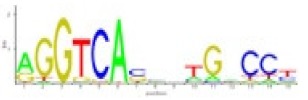
*FABP1*	AGCCAAGGTCATAGTGATGG	—
*FABP3*	GAGCCAGGGCTGAGTGCCCA	GTGTCACCCAGACAT
	AGACATGATCACTTTGACCC	—
*FABP6*	AAGTCAGATGACGATGCCCT	—
*FABP10*	—	AGGTCAGCAACCCCT

**Table 2:** Putative PPREs in chicken *FABP* genes promoter.

	5’FR ^a^			DR1 ^a^			
	Sequence	Fraction Similar	%Similar	Sequence	Fraction Similar	%Similar	Predicted PPAR Selectivity
Symbols	CAAAC			AGGTCANAGGTCA			
*FABP1*	GAAGT	3/5	0.6	GGACTATGGATTA	9.5/13	0.73	α
*FABP3*	GTGCT	1/5	0.2	CGGTATGAGGACA	9/13	0.69	γ
*FABP4*	AGAAC	3.5/5	0.7	GGGCCAAACTTCA	10/13	0.76	α
*FABP5*	AACAT	2.5/5	0.5	GAATTAGTGATCA	10/13	0.76	γ
*FABP6*	AAACT	2.5/5	0.5	GAATTGAAAGTGA	9/13	0.69	γ
*FABP7*	AAACT	2.5/5	0.5	AATTCTGAAAATA	8.5/13	0.65	γ
*FABP10*	GAATT	2.5/5	0.5	AGAGCACAAGTTT	10/13	0.76	γ

^a^ Fraction similarity is calculated based on the consensus of sequences. Briefly, the nucleotides complete matching were assigned a score of ‘1’, and the score of conservative substitution is 0.5 (i.e., purine to purine and pyrimidine to pyrimidine) [49]. Otherwise, if the similarity (% similar) of 5’FR > 0.5, it is judged to be regulated by PPARα, otherwise it is related to PPARγ; if the DR1 sequence is greater than 0.5, it is judged to be regulated by PPAR.

## Supplementary Materials

Supplementary materials can be found at https://www.mdpi.com/1422-0067/20/23/5948/s1.

## Abbreviations

*FABP*	fatty acid-binding protein
ER	estrogen receptor
PPAR	peroxisome proliferator activated receptor
FAs	Fatty acids
LCFAs	long-chain fatty acids
*iLBP*	intracellular lipid-binding protein
*CRABPs*	cellular retinoic acid binding proteins
*CRBPs*	cellular retinol-binding proteins
BAs	bile acids
TG	triacylglycerol
PPREs	peroxisome proliferator response elements
5′FR	5′ flanking region
DR1	direct repeat element
RXR	retinoid X receptor
ERE	estrogen response elements
*ACSF1*	Acyl-CoA synthetases family 1
MYA	millions of years ago
GFF	general feature format
TSS	transcript start sites
qPCR	quantitative PCR
MPP	1,3-bis(4-hydroxyphenyl)-4-methyl-5-[4-(2-piperidinylethoxy)phenol]-1*H*-pyrazoledihydrochloride
TAM	tamoxifen
ICI	ICI 182,780
cDNA	complementary DNA
aa	amino acid
MW	molecular weights
pIs	isoelectric points
CDS	coding sequence
UTR	untranslated region
*ACOX1*	acyl-coenzyme A oxidase 1
*PDK4*	pyruvate dehydrogenase kinase-4
*VCAM-1*	vascular cell adhesion molecule 1
WGD	whole genome duplication
MRCA	most recent common ancestor
h	hours

## References

[1] Donnelly K.L., Smith C.I., Schwarzenberg S.J., Jose J., Boldt M.D., Parks E.J. (2005). Sources of fatty acids stored in liver and secreted via lipoproteins in patients with nonalcoholic fatty liver disease. J. Clin. Investig..

[2] Mcarthur M.J., Atshaves B.P., Frolov A., Foxworth W.D., Kier A.B., Schroeder F. (1999). Cellular uptake and intracellular trafficking of long chain fatty acids. J. Lipid Res..

[3] Glatz J.F.C., Vusse G.J.V.D. (1996). Cellular fatty acid-binding proteins: Their function and physiological significance. Prog. Lipid Res..

[4] Lücke C., Gutiérrez-González L.H., Hamilton J.A., Asim K.D., Friedrich S. (2003). Intracellular lipid binding proteins: Evolution, structure, and ligand binding. Cellular Proteins and their Fatty Acids in Health and Disease.

[5] Parmar M.B., Venkatachalam A.B., Wright J.M. (2012). The evolutionary relationship of the transcriptionally active FABP11a (intronless) and *FABP11*b genes of medaka with FABP11 genes of other teleost fishes. FEBS J..

[6] Smathers R.L., Petersen D.R. (2011). The human fatty acid-binding protein family: Evolutionary divergences and functions. Hum. Genom..

[7] Sweetser D.A., Birkenmeier E.H., Klisak I.J., Zollman S., Sparkes R.S., Mohandas T., Lusis A.J., Gordon J.I. (1987). The human and rodent intestinal fatty acid binding protein genes. A comparative analysis of their structure, expression, and linkage relationships. J. Biol. Chem..

[8] Teruo O., Shoji O. (2010). Initial studies of the cytoplasmic FABP superfamily. Proc. Jpn. Acad..

[9] Chmurzyńska A. (2006). The multigene family of fatty acid-binding proteins (FABPs): Function, structure and polymorphism. J. Appl. Genet..

[10] Storch J., Corsico B. (2008). The emerging functions and mechanisms of mammalian fatty acid-binding proteins. Annu. Rev. Nutr..

[11] Schleicher C.H., Córdoba O.L., Santomé J.A., Dell’Angelica E.C. (1995). Molecular evolution of the multigene family of intracellular lipid-binding proteins. Biochem. Mol. Biol. Int..

[12] Judith S., Thumser A.E. (2010). Tissue-specific functions in the fatty acid-binding protein family. J. Biol. Chem..

[13] Spann N.J., Sohye K., Li A.C., Chen A.Z., Newberry E.P., Davidson N.O., Hui S.T.Y., Davis R.A. (2006). Coordinate transcriptional repression of liver fatty acid-binding protein and microsomal triglyceride transfer protein blocks hepatic very low density lipoprotein secretion without hepatosteatosis. J. Biol. Chem..

[14] Schievano E., Mammi S., Peggion E. (2015). Determination of the secondary structural elements of chicken liver fatty acid binding protein by two-dimensional homonuclear NMR. Biopolymers.

[15] Mukai T., Egawa M., Takeuchi T., Yamashita H., Kusudo T. (2017). Silencing of FABP1 ameliorates hepatic steatosis, inflammation and oxidative stress in mice with non-alcoholic fatty liver disease. FEBS Open Bio.

[16] Newberry E.P., Xie Y., Kennedy S.M., Luo J., Davidson N.O. (2010). Protection against Western diet–induced obesity and hepatic steatosis in liver fatty acid–binding protein knockout mice. Hepatology.

[17] Atshaves B.P., McIntosh A.L., Storey S.M., Landrock K.K., Kier A.B., Schroeder F. (2010). High dietary fat exacerbates weight gain and obesity in female liver fatty acid binding protein gene-ablated mice. Lipids.

[18] Shi H., Wang Q., Zhang Q., Leng L., Li H. (2010). Tissue expression characterization of chicken adipocyte fatty acid-binding protein and its expression difference between fat and lean birds in abdominal fat tissue. Poult. Sci..

[19] Agellon L.B., Drozdowski L., Li L., Iordache C., Luong L., Clandinin M.T., Uwiera R.R.E., Toth M.J., Thomson A.B.R. (2007). Loss of intestinal fatty acid binding protein increases the susceptibility of male mice to high fat diet-induced fatty liver. Biochim. Biophys. Acta.

[20] Angel A., Bray G.A. (2010). Synthesis of fatty acids and cholesterol by liver, adipose tissue and intestinal mucosa from obese and control patients. Eur. J. Clin. Investig..

[21] He Y., Yang X., Wang H., Estephan R., Francis F., Kodukula S., Storch J., Stark R.E. (2007). Solution-state molecular structure of apo and oleate-liganded liver fatty acid-binding protein. Biochemistry.

[22] Vassileva G. (2000). The intestinal fatty acid binding protein is not essential for dietary fat absorption in mice. FASEB J..

[23] Hughes A.L., Piontkivska H. (2011). Evolutionary diversification of the avian fatty acid-binding proteins. Gene.

[24] Murai A., Furuse M., Kitaguchi K., Kusumoto K., Nakanishi Y., Kobayashi M., Horio F. (2009). Characterization of critical factors influencing gene expression of two types of fatty acid-binding proteins (L-FABP and Lb-FABP) in the liver of birds. Comp. Biochem. Physiol. Part A Mol. Integr. Physiol..

[25] Wang Q., Li H., Li N., Leng L., Wang Y. (2006). Tissue expression and association with fatness traits of liver fatty acid-binding protein gene in chicken. Poult. Sci..

[26] Zhang Y., Liu Z., Liu R., Wang J., Zheng M., Li Q., Cui H., Zhao G., Wen J. (2018). Alteration of hepatic gene expression along with the inherited phenotype of acquired fatty liver in chicken. Genes.

[27] Wang Q., Li H., Liu S., Wang G., Wang Y. (2005). Cloning and tissue expression of chicken heart fatty acid-binding protein and intestine fatty acid-binding protein genes. Anim. Biotechnol..

[28] Li W.J., Li H.J., Zhao G.P., Zheng M.Q., Wen J. (2008). Gene expression of heart- and adipocyte-fatty acid-binding protein and correlation with intramuscular fat in Chinese chickens. Anim. Biotechnol..

[29] Chen M.C., Chang J.P., Lin Y.S., Pan K.L., Ho W.C., Liu W.H., Chang T.H., Huang Y.K., Fang C.Y., Chen C.J. (2016). Deciphering the gene expression profile of peroxisome proliferator-activated receptor signaling pathway in the left atria of patients with mitral regurgitation. J. Transl. Med..

[30] Wang Y., Mu Y., Li H., Ding N., Wang Q., Wang Y., Wang S., Wang N. (2008). Peroxisome proliferator-activated receptor-gamma gene: A key regulator of adipocyte differentiation in chickens. Poult. Sci..

[31] Sato K., Yonemura T., Ishii H., Toyomizu M., Kamada T., Akiba Y. (2009). Role of peroxisome proliferator-activated receptor β/δ in chicken adipogenesis. Comp. Biochem. Physiol. Part A Mol. Integr. Physiol..

[32] Laprairie R.B., Denovan-Wright E.M., Wright J.M. (2016). Subfunctionalization of peroxisome proliferator response elements accounts for retention of duplicated FABP1 genes in zebrafish. BMC Evol. Biol..

[33] Ricote M., Glass C.K. (2007). PPARs and molecular mechanisms of transrepression. Biochim. Biophys. Acta Biomembr..

[34] Christian S., Tanja E., Bertram B., Anton S., Friedrich S. (2004). Functional analysis of peroxisome-proliferator-responsive element motifs in genes of fatty acid-binding proteins. Biochem. J..

[35] Tan N.S., Shaw N.S., Vinckenbosch N., Peng L., Yasmin R., Desvergne B., Wahli W., Noy N. (2002). Selective cooperation between fatty acid binding proteins and peroxisome proliferator-activated receptors in regulating transcription. Mol. Cell. Biol..

[36] Pascual G., Glass C.K. (2006). Nuclear receptors versus inflammation: Mechanisms of transrepression. Trends Endocrinol. Metab..

[37] O’Hea E.K., Leveille G.A. (1969). Lipid biosynthesis and transport in the domestic chick (*Gallus domesticus*). Comp. Biochem. Physiol..

[38] Etches R.J., Cheng K.W. (1981). Changes in the plasma concentrations of luteinizing hormone, progesterone, oestradiol and testosterone and in the binding of follicle-stimulating hormone to the theca of follicles during the ovulation cycle of the hen (*Gallus domesticus*). J. Endocrinol..

[39] Beck M.M., Hansen K.K. (2004). Role of estrogen in avian osteoporosis. Poult. Sci..

[40] Tanabe Y., Nakamura T., Tanase H., Doi O. (1981). Comparisons of plasma LH, progesterone, testosterone and estradiol concentrations in male and female chickens (*Gallus domesticus*) from 28 to 1141 days of age. Endocrinol. Jpn..

[41] Kumar A., Bean L.A., Rani A., Jackson T., Foster T.C. (2016). Contribution of estrogen receptor subtypes, ERα, ERβ, and GPER1 in rapid estradiol-mediated enhancement of hippocampal synaptic transmission in mice. Hippocampus.

[42] Maria M., Paola G., Paolo A. (2006). Estrogen signaling multiple pathways to impact gene transcription. Curr. Genom..

[43] Carroll J.S., Meyer C.A., Jun S., Wei L., Geistlinger T.R., Jérôme E., Brodsky A.S., Erika Krasnickas K., Fertuck K.C., Hall G.F. (2006). Genome-wide analysis of estrogen receptor binding sites. Nat. Genet..

[44] Tian W., Zheng H., Yang L., Li H., Tian Y., Wang Y., Lyu S., Brockmann G.A., Kang X., Liu X. (2018). Dynamic expression profile, regulatory mechanism and correlation with egg-laying performance of ACSF gene family in chicken (*Gallus gallus*). Sci. Rep..

[45] Zheng H., Li H., Tan W., Xu C., Jia L., Wang D., Li Z., Sun G., Kang X., Yan F. (2018). Oestrogen regulates the expression of cathepsin E-A-like gene through ERβ in liver of chicken (*Gallus gallus*). J. Genet..

[46] Duan R., Ginsburg E., Vonderhaar B.K. (2008). Estrogen stimulates transcription from the human prolactin distal promoter through AP1 and estrogen responsive elements in T47D human breast cancer cells. Mol. Cell. Endocrinol..

[47] Petz L.N., Ziegler Y.S., Schultz J.R., Nardulli A.M. (2004). Fos and Jun inhibit estrogen-induced transcription of the human progesterone receptor gene through an activator protein-1 site. Mol. Endocrinol..

[48] Schaap F.G., Vusse G.J.V.D., Glatz J.F.C. (2002). Evolution of the family of intracellular lipid binding proteins in vertebrates. Mol. Cell. Biochem..

[49] Laprairie R.B., Denovan-Wright E.M., Wright J.M. (2017). Differential regulation of the duplicated, fabp7, fabp10, and, fabp11, genes of zebrafish by peroxisome proliferator activated receptors. Comp. Biochem. Physiol. Part B Biochem. Mol. Biol..

[50] Beigneux A.P., Moser A.H., Shigenaga J.K., Grunfeld C., Feingold K.R. (2000). The acute phase response is associated with retinoid X receptor repression in rodent liver. J. Biol. Chem..

[51] Kuo S.C., Ku P.M., Chen L.J., Niu H.S., Cheng J.T. (2013). Activation of receptors delta (PPAR delta) by agonist (GW0742) may enhance lipid metabolism in heart both in vivo and in vitro. Horm. Metab. Res..

[52] Sánchezgurmaches J., Cruzgarcia L., Gutiérrez J., Navarro I. (2012). mRNA expression of fatty acid transporters in rainbow trout: In vivo and in vitro regulation by insulin, fasting and inflammation and infection mediators. Comp. Biochem. Physiol. Part A Mol. Integr. Physiol..

[53] Paramvir D., Boore J.L. (2005). Two rounds of whole genome duplication in the ancestral vertebrate. PLoS Biol..

[54] Yves V.D.P., Steven M., Axel M. (2010). 2R or not 2R is not the question anymore. Nat. Rev. Genet..

[55] Venkatachalam A.B., Parmar M.B., Wright J.M. (2017). Evolution of the duplicated intracellular lipid-binding protein genes of teleost fishes. Mol. Genet. Genom..

[56] Venkatachalam A.B., Fontenot Q., Farrara A., Wright J.M. (2017). Fatty acid-binding protein genes of the ancient, air-breathing, ray-finned fish, spotted gar (*Lepisosteus oculatus*). Comp. Biochem. Physiol. Part D Genom. Proteom..

[57] Robinson-Rechavi M., Marchand O., Escriva H., Bardet P.L., Zelus D., Hughes S., Laudet V. (2001). Euteleost fish genomes are characterized by expansion of gene families. Genome Res..

[58] Glasauer S.M.K., Neuhauss S.C.F. (2014). Whole-genome duplication in teleost fishes and its evolutionary consequences. Mol. Genet. Genom..

[59] Raes J. (2004). Duplication and divergence: The evolution of new genes and old ideas. Annu. Rev. Genet..

[60] Kumar S., Hedges S.B. (1998). A molecular timescale for vertebrate evolution. Nature.

[61] Ockner R.K., Manning J.A., Poppenhausen R.B. (1972). A binding protein for fatty acids in cytosol of intestinal mucosa, liver, myocardium, and other tissues. Science.

[62] Storch J., Mcdermott L. (2009). Structural and functional analysis of fatty acid-binding proteins. J. Lipid Res..

[63] Linjie W., Li L., Jing J., Yan W., Tao Z., Yu C., Yong W., Hongping Z. (2015). Molecular characterization and different expression patterns of the FABP gene family during goat skeletal muscle development. Mol. Biol. Rep..

[64] Liu R.Z., Li X., Godbout R. (2008). A novel fatty acid-binding protein (FABP) gene resulting from tandem gene duplication in mammals: Transcription in rat retina and testis. Genomics.

[65] Schneider W. (2016). Lipid transport to avian oocytes and to the developing embryo. J. Biomed. Res..

[66] Kuiper G.G., Carlsson B., Grandien K., Enmark E., Häggblad J., Nilsson S., Gustafsson J.A. (1997). Comparison of the ligand binding specificity and transcript tissue distribution of estrogen receptors alpha and beta. Endocrinology.

[67] Palmer C.N., Hsu M.H., Griffin H.J., Johnson E.F. (1995). Novel sequence determinants in peroxisome proliferator signaling. J. Biol. Chem..

[68] Qi C., Zhu Y., Reddy J.K. (2000). Peroxisome proliferator-activated receptors, coactivators, and downstream targets. Cell Biochem. Biophys..

[69] Dubois V., Jérôme E., Lefebvre P., Staels B. (2017). Distinct but complementary contributions of PPAR isotypes to energy homeostasis. J. Clin. Investig..

[70] Rondón-Ortiz A.N., Cardenas C.L.L., Martínez-Málaga J., Gonzales-Urday A.L., Gugnani K.S., Böhlke M., Maher T.J., Pino-Figueroa A.J. (2017). High Concentrations of rosiglitazone reduce mRNA and protein levels of LRP1 in HepG2 cells. Front. Pharmacol..

[71] Lee H., Yoon M. (2013). 17β-estradiol inhibits PPARα of skeletal muscle. Anim. Cells Syst..

[72] Madureira T.V., Pinheiro I., Malhão F., Castro L.F.C., Rocha E., Urbatzka R. (2018). Silencing of PPARαBb mRNA in brown trout primary hepatocytes: Effects on molecular and morphological targets under the influence of an estrogen and a PPARα agonist. Comp. Biochem. Physiol. Part B Biochem. Mol. Biol..

[73] Jeong S., Yoon M. (2012). Inhibition of the actions of peroxisome proliferator-activated receptor α on obesity by estrogen. Obesity.

[74] Johnson L.S., Eddy S.R., Portugaly E. (2010). Hidden Markov model speed heuristic and iterative HMM search procedure. BMC Bioinform..

[75] Altschul S.F. (1990). Basic local alignment search tool (BLAST). J. Mol. Biol..

[76] Strimmer K., Haeseler A.V. (1996). Quartet puzzling: A quartet maximum-likelihood method for reconstructing tree topologies. Mol. Biol. Evol..

[77] Aiyar A. (2000). The use of CLUSTAL W and CLUSTAL X for multiple sequence alignment. Methods Mol. Biol..

[78] Styczynski M.P., Jensen K.L., Rigoutsos I., Stephanopoulos G. (2008). BLOSUM62 miscalculations improve search performance. Nat. Biotechnol..

[79] Kumar S., Stecher G., Tamura K. (2016). MEGA7: Molecular evolutionary genetics analysis version 7.0 for bigger datasets. Mol. Biol. Evol..

[80] Koichiro T., Fabia Ursula B., Paul B.R., Oscar M., Alan F., Sudhir K. (2012). Estimating divergence times in large molecular phylogenies. Proc. Natl. Acad. Sci. USA.

[81] Jones D.T., Taylor W.R., Thornton J.M. (1992). The rapid generation of mutation data matrices from protein sequences. Comput. Appl. Biosci..

[82] Chen C., Chen H., He Y., Xia R. (2018). TBtools, a Toolkit for Biologists integrating various HTS-data handling tools with a user-friendly interface. BioRxiv.

[83] Ren J., Xu N., Ma Z., Li Y., Li C., Wang Y., Tian Y., Liu X., Kang X., Ryan A. (2018). Characteristics of expression and regulation of sirtuins in chicken (*Gallus gallus*). J. Agric. Biotechnol..

[84] Begam A.J., Jubie S., Nanjan M.J. (2017). Estrogen receptor agonists/antagonists in breast cancer therapy: A critical review. Bioorg. Chem..

[85] Kawaguchi T., Nomura K., Hirayama Y., Kitagawa T. (1987). Establishment and characterization of a chicken hepatocellular carcinoma cell line, LMH. Cancer Res..

