# Supplementary material for: Genome-Wide Analysis of the FABP Gene Family in Liver of Chicken (Gallus gallus): Identification, Dynamic Expression Profile, and Regulatory Mechanism
Source: Int J Mol Sci. 2019 Nov 26;20(23):5948. doi: 10.3390/ijms20235948 (PMC6928644; doi:10.3390/ijms20235948)
Supplement: Supplementary file 1 [file ijms-20-05948-s001.zip › supplementary material.doc]

**Supplementary Data**

**Supplementary Fig S1.** Experimental design of this research.

**Supplementary Fig S2.** Sequence alignment of chicken FABP proteins. Horizontal line specify amino acid identity and dashes represent gaps. The percentage sequence identity and similarity of chicken FABP sequences are shown at the end of each sequence.

**Supplementary Fig S3.** Predicted regulatory elements in chicken *FABP* gene promoters. Promoter sequences (−2000 bp) of nine *FABP* genes are analyzed by JASPAR and MEME software. The upstream length to the translation start site can be inferred according to the scale at the bottom.

**Supplementary Fig S4.** *FABP* gene family members which expressed in chicken liver are regulated by estrogen and PPAR signaling pathways. The red and blue boxes represent the predicted ERE and PPRE site alone in the promoter of chicken *FABP* genes, respectively.

**Supplementary Fig S5.** PPARs and Estrogen regulate the expression of *FABPs* in chicken hepatocytes.

**Supplementary table S1. Features of *FABP* genes identified in chicken**

| **Symbol** | **Common name** | **Chr.** | **Genomic Location** | **ORF** | **AA** | **MW (kDa)** | **PI** |
| --- | --- | --- | --- | --- | --- | --- | --- |
| FABP1 | Fatty acid-binding protein 1, liver | 4 | 85,982,308-85,986,038 | 384 | 127 | 14192.35 | 7.73 |
| FABP2 | Fatty acid-binding protein 2, intestinal | 4 | 54,716,756-54,719,201 | 399 | 132 | 15089.16 | 6.61 |
| FABP3 | Fatty acid-binding protein 3, heart | 23 | 703,869-706,870 | 402 | 133 | 14843.96 | 5.92 |
| FABP4 | Fatty acid-binding protein 4, adipocyte | 2 | 121,350,127-121,353,349 | 399 | 132 | 14921.00 | 6.34 |
| FABP5 | Fatty acid-binding protein 5, epidermal | 2 | 121,272,966-121,278,024 | 405 | 134 | 15079.43 | 5.59 |
| FABP6 | Fatty acid-binding protein 6, ileal | 13 | 8,581,097-8,631,647 | 348 | 115 | 12864.50 | 5.33 |
| FABP7 | Fatty acid-binding protein 7, brain | 3 | 61,282,801-61,286,154 | 399 | 132 | 14926.98 | 5.61 |
| FABP8 | Peripheral myelin protein 2 | 2 | 121,340,221-121,344,668 | 399 | 132 | 14844.30 | 9.45 |
| FABP10 | Liver basic fatty acid binding protein | 23 | 5,776,686-5,779,201 | 381 | 126 | 14212.40 | 8.53 |

**Supplementary table S2.** List of the conserved FATTYACIDBP domain of chicken FABP proteins

| **Symbol** | **FATTYACIDBP1 (%)** | **FATTYACIDBP2 (%)** | **FATTYACIDBP3 (%)** |
| --- | --- | --- | --- |
| FABP1 | FSGTWQVYAQENYEEFLKALALP | SFTLGKEADITTMDGKK | KGNEMVETITFGGVTLIRRSK |
| FABP2 | FNGTWKIEKNENYEKFMEAMGVN (43.48) | EFTLGVSFEYSLADGTE (35.29) | GSELIQTYVYEGVEAKRIFKK (31.82) |
| FABP3 | FVGTWKLVDTANFDEYMKALGVG (39.13) | SFKLDEEFDETTADDRH (47.06) | DGKLILTLTMGNVVSTRTYEK (27.27) |
| FABP4 | FVGTWKLLSSENFEDYMKELGVG (39.13) | SFKLGEEFDETTADDRK (58.82) | DGNLLVECTMNNVTSKRVYER (36.36) |
| FABP5 | FLGKWCLVSSEGFEDYMKELGVG (30.43) | SFKIGEKFEENTLDGRK (41.18) | DGQLVVECDMNGVKCVRVYQK (22.73) |
| FABP6 | FTGKYEFESDENYDDFVKKIGLP (39.13) | SFTIDKEADMETMGGRK (64.71) | GGKLVEVMSV (14.29) |
| FABP7 | FCATWKLADSHNFDEYMKALGVG (34.78) | SFKLGEEFDETTPDDRN (52.94) | DGRMVMTLTFGDVVAVRHYEK (40.91) |
| FABP8 | FVGTWKLVSSENFDDYMKELGVG (34.78) | SFKLGQQFDETTADDRK (52.94) | DGKMVVECAMKGVVCTRVYER (27.27) |
| FABP10 | FTGKYELQSHENFEPFMKALGLP (47.83) | EFTIGEECEMELLTGEK (35.29) | GDTITHTMTKGDLTYKRISKR (36.36) |

**Note**: Sequence alignment of FATTYACIDBP domain among chicken FABP pretoins.

**Supplementary table S3.** List of the exon numbers of chicken *FABP* genes

| **Symbol** | **EXON1 (aa)** | **EXON2 (aa)** | **EXON3 (aa)** | **EXON4 (aa)** | **EXON5 (aa)** | **EXON6 (aa)** |
| --- | --- | --- | --- | --- | --- | --- |
| FABP1 | 33 | 58 | 42 | 16 |  |  |
| FABP2 | 22 | 57 | 35 | 16 |  |  |
| FABP3 | 24 | 57 | 42 | 17 |  |  |
| FABP4 | 24 | 57 | 34 | 16 |  |  |
| FABP5 | 25 | 57 | 34 | 16 |  |  |
| FABP6 | 33 | 58 | 30 | 17 | 30 | 46 |
| FABP7 | 24 | 57 | 33 | 16 |  |  |
| FABP9 | 24 | 57 | 33 | 16 |  |  |
| FABP10 | 33 | 57 | 30 | 16 |  |  |

**Supplementary table S4.** List of the twelve FABP protein sequences as a qurey template to build multiple alignment model

| **Species** | **Symbol** | **NCBI accession numbers** |
| --- | --- | --- |
| Human | FABP1 | [NP_001434.1](https://www.ncbi.nlm.nih.gov/protein/NP_001434.1) |
|  | FABP2 | [NP_000125.2](https://www.ncbi.nlm.nih.gov/protein/NP_000125.2) |
|  | FABP3 | [NP_004093.1](https://www.ncbi.nlm.nih.gov/protein/NP_004093.1) |
|  | FABP4 | [NP_001433.1](https://www.ncbi.nlm.nih.gov/protein/NP_001433.1) |
|  | FABP5 | [NP_001435.1](https://www.ncbi.nlm.nih.gov/protein/NP_001435.1) |
|  | FABP6 | [NP_001035532.1](https://www.ncbi.nlm.nih.gov/protein/NP_001035532.1) |
|  | FABP7 | [NP_001305968.1](https://www.ncbi.nlm.nih.gov/protein/NP_001305968.1) |
|  | FABP8 | [NP_001335310.1](https://www.ncbi.nlm.nih.gov/protein/NP_001335310.1) |
|  | FABP9 | [NP_001073995.1](https://www.ncbi.nlm.nih.gov/protein/NP_001073995.1) |
| Zebrafish | FABP10a | [NP_694492.1](https://www.ncbi.nlm.nih.gov/protein/NP_694492.1) |
|  | FABP10b | [XP_017207372.1](https://www.ncbi.nlm.nih.gov/protein/XP_017207372.1) |
|  | FABP11a | [NP_001004682.1](https://www.ncbi.nlm.nih.gov/protein/NP_001004682.1) |
|  | FABP11b | [NP_001018394.1](https://www.ncbi.nlm.nih.gov/protein/NP_001018394.1) |
| Human | FABP12 | [NP_001098751.1](https://www.ncbi.nlm.nih.gov/protein/NP_001098751.1) |

**Supplementary table S5.** Primers used in this study

| **Gene** | **Primer name** | **Primer sequences** | **Tm (℃)** |
| --- | --- | --- | --- |
| FABP1 | FABP1-F | TCTGAAAGCTCTTGCACTGCC | 60 |
|  | FABP1-R | TCACAGTCTGCCTGGGTGTT |  |
| FABP2 | FABP2-F | TCATGGAAGCAATGGGCGTG | 60 |
|  | FABP2-R | TCCCGTCAGCCAGACTGTAT |  |
| FABP3 | FABP3-F | GAGTTCGATGAGACCACGGC | 60 |
|  | FABP3-R | CGTTGCCCATGGTGAGAGTC |  |
| FABP4 | FABP4-F | GCACCTGGAAGCTCCTTTCT | 60 |
|  | FABP4-R | ATTAGGCTTGGCCACACCAG |  |
| FABP5 | FABP5-F | TGGCCATCGACGCGTTTTTA | 60 |
|  | FABP5-R | TCTGGTTTCGCCATGCTTCC |  |
| FABP6 | FABP6-F | CCACCCATTGTCTCCATGTC | 60 |
|  | FABP6-R | CCCTGCTGACAAGATCGAAA |  |
| FABP7 | FABP7-F | GTGGGGAACGTGACTAAGCC | 60 |
|  | FABP7-R | GCTTGTCTCCATCCAGGGTC |  |
| FABP8 | FABP8-F | AGCCTGGTTACCTGGGACAA | 60 |
|  | FABP8-R | TCCTTCGAGCCAGTGAGTCC |  |
| FABP10 | FABP10-F | ACGAGCTCCAGTCCCATGAA | 60 |
|  | FABP10-R | TCCATCTCACACTCTTCCCCA |  |
| ACOX1 | ACOX1-F | GCCAGGTGGACTTGGAAAGA | 60 |
|  | ACOX1-R | GCTGCCGTATAGGAACAATGAAG |  |
| VCAM-1 | VCAM-F | TCCCACTTGCCTCTCCCTAA | 60 |
|  | VCAM-R | CCTCCCGGTAGGCAAACTG |  |
| PDK4 | PDK4-F | GCTTTTACATGAGCCGCATTT | 60 |
|  | PDK4-R | CAACAACATCACAGCAAGGATCA |  |
| β-action | β-action-F | CAGCCAGCCATGGATGATGA | 60 |
|  | β-action-R | ACCAACCATCACACCCTGAT |  |
| PPAR α | PPAR α-F | GGAGACCTTAAGTGACACCCTCT | 60 |
|  | PPAR α-R | CTGATCCATCAGATCCTGGA |  |
| PPAR β | PPAR β-F | CTTGCTGAGCCGCACCACTC | 60 |
|  | PPAR β-R | TCACACCCGCCAAGGCAAGAT |  |
| PPAR γ | PPAR γ-F | CTCCTTCTCCTCCCTATTT | 60 |
|  | PPAR γ-R | TTTCTTATGGATGCGACA |  |
